# Supplementary material for: Standardised Outcome Reporting for the Nutrition Management of Complex Chronic Disease: A Rapid Review
Source: Nutrients. 2021 Sep 26;13(10):3388. doi: 10.3390/nu13103388 (PMC8538850; doi:10.3390/nu13103388)

Supplementary Materials

Standardised outcome reporting for the nutrition management of complex chronic disease: A rapid review

Savita A Sandhu[1*], Chloe A Angel[1*], Katrina L Campbell[2], Ingrid J Hickman[3,4], Helen L MacLaughlin[1,5]

[1] School of Exercise and Nutrition Sciences, Queensland University of Technology (QUT); savita.sandhu@connect.qut.edu.au; chloe.angel@connect.qut.edu.au
[2] Healthcare Excellence and Innovation, Metro North Hospital and Health Service, Brisbane, Australia; katrina.campbell@health.qld.gov.au
[3] Department of Nutrition and Dietetics, Princess Alexandra Hospital, Brisbane Australia; i.hickman@uq.edu.au
[4] Faculty of Medicine, University of Queensland, Brisbane, Australia
[5] Royal Brisbane and Women's Hospital, Brisbane, Australia; h.maclaughlin@qut.edu.au
* equal co-authorship

Table of Contents

Prisma 2020 for Abstracts Checklist	…………………………………………………..	3
Prisma 2020 Checklist	…………………………………………………..	4
Supplementary Materials RCT Database Search Strategies	…………………………………………………..	7
Supplementary Materials Figure S1: Frequency and number of outcomes and outcome measures reported by RCTs	…………………………………………………..	9
Supplementary Materials Figure S2: Outcome measures for change in body composition reported by RCTs	…………………………………………………..	10
Supplementary Material Figure S3: Outcome measures for glycaemic control reported by RCTs	…………………………………………………..	10
Supplementary Material Figure S4: Outcome measures for change in lipid profile reported by RCTs	…………………………………………………..	11
Supplementary Material Figure S5: Outcome measures for change in dietary intake reported by RCT	…………………………………………………..	12
Supplementary Material Figure S6: Quality assessment of RCT using Cochrane Risk of Bias 2.0 Tool	…………………………………………………..	13

Prisma 2020 for Abstracts Checklist

Section and Topic	Item #	Checklist item	Reported (Yes/No)
**TITLE**			
Title	1	Identify the report as a systematic review.	No
**BACKGROUND**			
Objectives	2	Provide an explicit statement of the main objective(s) or question(s) the review addresses.	Yes
**METHODS**			
Eligibility criteria	3	Specify the inclusion and exclusion criteria for the review.	Yes
Information sources	4	Specify the information sources (e.g. databases, registers) used to identify studies and the date when each was last searched.	Yes
Risk of bias	5	Specify the methods used to assess risk of bias in the included studies.	Yes
Synthesis of results	6	Specify the methods used to present and synthesise results.	Yes
**RESULTS**			
Included studies	7	Give the total number of included studies and participants and summarise relevant characteristics of studies.	Yes
Synthesis of results	8	Present results for main outcomes, preferably indicating the number of included studies and participants for each. If meta-analysis was done, report the summary estimate and confidence/credible interval. If comparing groups, indicate the direction of the effect (i.e. which group is favoured).	Yes
**DISCUSSION**			
Limitations of evidence	9	Provide a brief summary of the limitations of the evidence included in the review (e.g. study risk of bias, inconsistency and imprecision).	Yes
Interpretation	10	Provide a general interpretation of the results and important implications.	Yes
**OTHER**			
Funding	11	Specify the primary source of funding for the review.	N/A
Registration	12	Provide the register name and registration number.	N/A

From: Page MJ, McKenzie JE, Bossuyt PM, Boutron I, Hoffmann TC, Mulrow CD, et al. The PRISMA 2020 statement: an updated guideline for reporting systematic reviews. BMJ 2021;372:n71. doi: 10.1136/bmj.n71

Prisma 2020 Checklist

Section and Topic	Item #	Checklist item	Page #
**TITLE**			
Title	1	Identify the report as a systematic review.	1
**ABSTRACT**			
Abstract	2	See the PRISMA 2020 for Abstracts checklist.	See above
**INTRODUCTION**			
Rationale	3	Describe the rationale for the review in the context of existing knowledge.	2
Objectives	4	Provide an explicit statement of the objective(s) or question(s) the review addresses.	2
**METHODS**			
Eligibility criteria	5	Specify the inclusion and exclusion criteria for the review and how studies were grouped for the syntheses.	3-5
Information sources	6	Specify all databases, registers, websites, organisations, reference lists and other sources searched or consulted to identify studies. Specify the date when each source was last searched or consulted.	3
Search strategy	7	Present the full search strategies for all databases, registers and websites, including any filters and limits used.	Supplementary Materials
Selection process	8	Specify the methods used to decide whether a study met the inclusion criteria of the review, including how many reviewers screened each record and each report retrieved, whether they worked independently, and if applicable, details of automation tools used in the process.	3
Data collection process	9	Specify the methods used to collect data from reports, including how many reviewers collected data from each report, whether they worked independently, any processes for obtaining or confirming data from study investigators, and if applicable, details of automation tools used in the process.	4
Data items	10a	List and define all outcomes for which data were sought. Specify whether all results that were compatible with each outcome domain in each study were sought (e.g. for all measures, time points, analyses), and if not, the methods used to decide which results to collect.	4
	10b	List and define all other variables for which data were sought (e.g. participant and intervention characteristics, funding sources). Describe any assumptions made about any missing or unclear information.	4
Study risk of bias assessment	11	Specify the methods used to assess risk of bias in the included studies, including details of the tool(s) used, how many reviewers assessed each study and whether they worked independently, and if applicable, details of automation tools used in the process.	5
Effect measures	12	Specify for each outcome the effect measure(s) (e.g. risk ratio, mean difference) used in the synthesis or presentation of results.	4-5
Synthesis methods	13a	Describe the processes used to decide which studies were eligible for each synthesis (e.g. tabulating the study intervention characteristics and comparing against the planned groups for each synthesis (item #5)).	4-5
	13b	Describe any methods required to prepare the data for presentation or synthesis, such as handling of missing summary statistics, or data conversions.	4-5
	13c	Describe any methods used to tabulate or visually display results of individual studies and syntheses.	4-5
	13d	Describe any methods used to synthesize results and provide a rationale for the choice(s). If meta-analysis was performed, describe the	4-5

Section and Topic	Item #	Checklist item	Page #
		model(s), method(s) to identify the presence and extent of statistical heterogeneity, and software package(s) used.	
	13e	Describe any methods used to explore possible causes of heterogeneity among study results (e.g. subgroup analysis, meta-regression).	N/A
	13f	Describe any sensitivity analyses conducted to assess robustness of the synthesized results.	N/A
Reporting bias assessment	14	Describe any methods used to assess risk of bias due to missing results in a synthesis (arising from reporting biases).	4
Certainty assessment	15	Describe any methods used to assess certainty (or confidence) in the body of evidence for an outcome.	N/A
**RESULTS**			
Study selection	16a	Describe the results of the search and selection process, from the number of records identified in the search to the number of studies included in the review, ideally using a flow diagram.	6
	16b	Cite studies that might appear to meet the inclusion criteria, but which were excluded, and explain why they were excluded.	6
Study characteristics	17	Cite each included study and present its characteristics.	7-8, 10
Risk of bias in studies	18	Present assessments of risk of bias for each included study.	4
Results of individual studies	19	For all outcomes, present, for each study: (a) summary statistics for each group (where appropriate) and (b) an effect estimate and its precision (e.g. confidence/credible interval), ideally using structured tables or plots.	N/A
Results of syntheses	20a	For each synthesis, briefly summarise the characteristics and risk of bias among contributing studies.	16
	20b	Present results of all statistical syntheses conducted. If meta-analysis was done, present for each the summary estimate and its precision (e.g. confidence/credible interval) and measures of statistical heterogeneity. If comparing groups, describe the direction of the effect.	N/A
	20c	Present results of all investigations of possible causes of heterogeneity among study results.	9, 11-16
	20d	Present results of all sensitivity analyses conducted to assess the robustness of the synthesized results.	N/A
Reporting biases	21	Present assessments of risk of bias due to missing results (arising from reporting biases) for each synthesis assessed.	N/A
Certainty of evidence	22	Present assessments of certainty (or confidence) in the body of evidence for each outcome assessed.	N/A
**DISCUSSION**			
Discussion	23a	Provide a general interpretation of the results in the context of other evidence.	16-19
	23b	Discuss any limitations of the evidence included in the review.	19
	23c	Discuss any limitations of the review processes used.	19
	23d	Discuss implications of the results for practice, policy, and future research.	19
**OTHER INFORMATION**			

Section and Topic	Item #	Checklist item	Page #
Registration and protocol	24a	Provide registration information for the review, including register name and registration number, or state that the review was not registered.	2
	24b	Indicate where the review protocol can be accessed, or state that a protocol was not prepared.	N/A
	24c	Describe and explain any amendments to information provided at registration or in the protocol.	N/A
Support	25	Describe sources of financial or non-financial support for the review, and the role of the funders or sponsors in the review.	19
Competing interests	26	Declare any competing interests of review authors.	19
Availability of data, code and other materials	27	Report which of the following are publicly available and where they can be found: template data collection forms; data extracted from included studies; data used for all analyses; analytic code; any other materials used in the review.	19

Supplementary Materials: RCT Database Search Strategies

PubMed

("Nutrition Therapy"[Mesh] OR Diet[Mesh] OR "nutrition" OR "nutritional" OR "diet" OR "dietary" OR "intake") AND ("Diabetes Mellitus, Type 2"[Mesh] OR t2dm OR ("diabetes" AND ("type-2" OR "type two" OR "type II" OR "type 2")) OR "Renal Insufficiency, Chronic"[Mesh] OR "Cardio-Renal Syndrome"[Mesh] OR CKD OR "renal insufficiency, chronic" OR "diabetic nephropathies" OR ("chronic renal" AND (insufficiency OR "disease" OR failure)) OR (("diabetic" OR chronic) AND "kidney disease") OR (("cardio-renal" OR "cardiorenal") AND "syndrome") OR "Heart Failure"[Mesh] OR "Coronary Disease"[Mesh] OR "Myocardial Ischemia"[Mesh] OR "Arteriosclerosis"[Mesh] OR "Stroke"[Mesh] OR "Carotid Artery Diseases"[Mesh] OR "Diabetic Angiopathies"[Mesh] OR ("myocardial" AND ("infarction" or "ischaemia" or "ischemia")) OR "mi" OR "pci" OR "cabg" OR "tia" OR "heart failure" OR "arteriosclerosis" OR "atherosclerosis" OR "stroke" OR "transient ischaemic attack" OR "diabetic angiopathies" OR "carotid stenosis" OR (("cardiovascular" OR "coronary" OR "heart" OR "carotid artery") AND "disease") OR ("coronary artery" AND ("disease" OR bypass OR "bypass graft")) OR "percutaneous coronary intervention" OR "Non-alcoholic Fatty Liver Disease"[Mesh] OR "nafld" OR "nash" OR "liver cirrhosis" OR (nonalcoholic AND ("fatty liver disease" OR "steatohepatitis")) OR ("non alcoholic" AND ("fatty liver disease" OR "steatohepatitis")) OR ("non-alcoholic" AND ("fatty liver disease" OR "steatohepatitis")) OR "Metabolic Syndrome"[Mesh] OR "Metabolic Syndrome" OR "Metabolic Syndrome X" OR "Syndrome X") AND (Randomized Controlled Trial[ptyp] AND ("2010/01/01"[PDAT] : "2020/12/31"[PDAT]) AND "humans"[MeSH Terms]).

Embase

('diet therapy'/exp OR 'nutrition'/exp OR 'diet'/exp OR diet*:ab,ti OR nutrition*:ab,ti OR intake:ab,ti) AND ('non insulin dependent diabetes'/exp OR 't2dm':ab,ti OR ('diabetes':ab,ti AND ('type-2':ab,ti OR 'type two':ab,ti OR 'type ii':ab,ti OR 'type 2':ab,ti)) OR 'chronic kidney failure'/exp OR 'cardiorenal syndrome'/exp OR 'diabetic nephropathy'/exp OR ckd:ab,ti OR 'renal insufficiency, chronic':ab,ti OR 'diabetic nephropathies':ab,ti OR ('chronic renal':ab,ti AND (insufficiency:ab,ti OR 'disease':ab,ti OR 'failure':ab,ti)) OR (('diabetic':ab,ti OR chronic:ab,ti) AND 'kidney disease':ab,ti) OR (('cardio-renal':ab,ti OR 'cardiorenal':ab,ti) AND 'syndrome':ab,ti) OR 'heart disease'/exp OR 'vascular disease'/exp OR 'cardiovascular symptom'/exp OR (myocardial:ab,ti AND (infarction:ab,ti OR ischaemia:ab,ti OR ischemia:ab,ti)) OR mi:ab,ti OR pci:ab,ti OR cabg:ab,ti OR tia:ab,ti OR 'heart failure':ab,ti OR arteriosclerosis:ab,ti OR atherosclerosis:ab,ti OR stroke:ab,ti OR 'transient ischaemic attack':ab,ti OR 'diabetic angiopathies':ab,ti OR 'carotid stenosis':ab,ti OR ((cardiovascular:ab,ti OR coronary:ab,ti OR heart:ab,ti OR 'carotid artery':ab,ti) AND disease:ab,ti) OR ('coronary artery':ab,ti AND (disease:ab,ti OR bypass:ab,ti OR 'bypass graft':ab,ti)) OR 'percutaneous coronary intervention':ab,ti OR 'nonalcoholic fatty liver'/exp OR 'nonalcoholic steatohepatitis'/exp OR nafld:ab,ti OR nash:ab,ti OR 'liver cirrhosis':ab,ti OR (nonalcoholic:ab,ti AND ('fatty liver disease':ab,ti OR steatohepatitis:ab,ti)) OR ('non alcoholic':ab,ti AND ('fatty liver disease':ab,ti OR steatohepatitis:ab,ti)) OR ('non-alcoholic':ab,ti AND ('fatty liver disease':ab,ti OR steatohepatitis:ab,ti)) OR 'metabolic syndrome':ab,ti OR 'metabolic syndrome'/de OR 'metabolic syndrome x':ab,ti OR 'syndrome x':ab,ti) NOT ('cohort' OR 'retrospective') AND [randomized controlled trial]/lim AND [article]/lim AND [humans]/lim AND [2010-2020]/py AND [embase]/lim

CINAHL

(((MH "Nutrition+") OR (MH "Diet+") OR (MH "intake")) OR (TI (nutrition* OR diet* OR intake*) OR AB (nutrition* OR diet* OR intake*))) AND (MH ("diabetes mellitus, type 2" OR "kidney failure, chronic"

OR "renal insufficiency, chronic" OR "diabetic nephropathies" OR "nonalchoholic fatty liver disease" OR "liver cirrhosis" OR "cardiovascular diseases+" OR "metabolic syndrome X") OR TI ("t2dm" OR ("diabetes" AND ("type-2" OR “type two” OR “type II” OR “type 2”)) OR "ckd" OR ("chronic renal" AND ("insufficiency" OR "disease" OR "failure")) OR (("diabetic" OR "chronic") AND "kidney disease") OR (("cardio-renal" OR "cardiorenal") AND "syndrome") OR ("myocardial" AND ("infarction" or "ischaemia" or "ischemia")) OR "mi" OR "pci" OR "cabg" OR "tia" OR "heart failure" OR "arteriosclerosis" OR "atherosclerosis" OR "stroke" OR "transient ischaemic attack" OR "diabetic angiopathies" OR "carotid stenosis" OR (("cardiovascular" OR "coronary" OR "heart" OR "carotid artery") AND "disease") OR ("coronary artery" AND ("disease" OR "bypass" OR "bypass graft")) OR "percutaneous coronary intervention" OR "nafld" OR "nash" OR "liver cirrhosis" OR ("nonalcoholic" AND ("fatty liver disease" OR "steatohepatitis")) OR ("non alcoholic" AND ("fatty liver disease" OR "steatohepatitis")) OR ("non-alcoholic" AND ("fatty liver disease" OR "steatohepatitis")) OR “Metabolic Syndrome” OR “Metabolic Syndrome X” OR “Syndrome X”) OR AB ("t2dm" OR ("diabetes" AND ("type-2" OR “type two” OR “type II” OR “type 2”)) OR "ckd" OR ("chronic renal" AND ("insufficiency" OR "disease" OR "failure")) OR (("diabetic" OR "chronic") AND "kidney disease") OR (("cardio-renal" OR "cardiorenal") AND "syndrome") OR ("myocardial" AND ("infarction" or "ischaemia" or "ischemia")) OR "mi" OR "pci" OR "cabg" OR "tia" OR "heart failure" OR "arteriosclerosis" OR "atherosclerosis" OR "stroke" OR "transient ischaemic attack" OR "diabetic angiopathies" OR "carotid stenosis" OR (("cardiovascular" OR "coronary" OR "heart" OR "carotid artery") AND "disease") OR ("coronary artery" AND ("disease" OR "bypass" OR "bypass graft")) OR "percutaneous coronary intervention" OR "nafld" OR "nash" OR "liver cirrhosis" OR ("nonalcoholic" AND ("fatty liver disease" OR "steatohepatitis")) OR ("non alcoholic" AND ("fatty liver disease" OR "steatohepatitis")) OR ("non-alcoholic" AND ("fatty liver disease" OR "steatohepatitis")) OR “Metabolic Syndrome” OR “Metabolic Syndrome X” OR “Syndrome X”)

Supplementary Materials Figure S1: Frequency and number of outcomes and outcome measures reported by RCTs

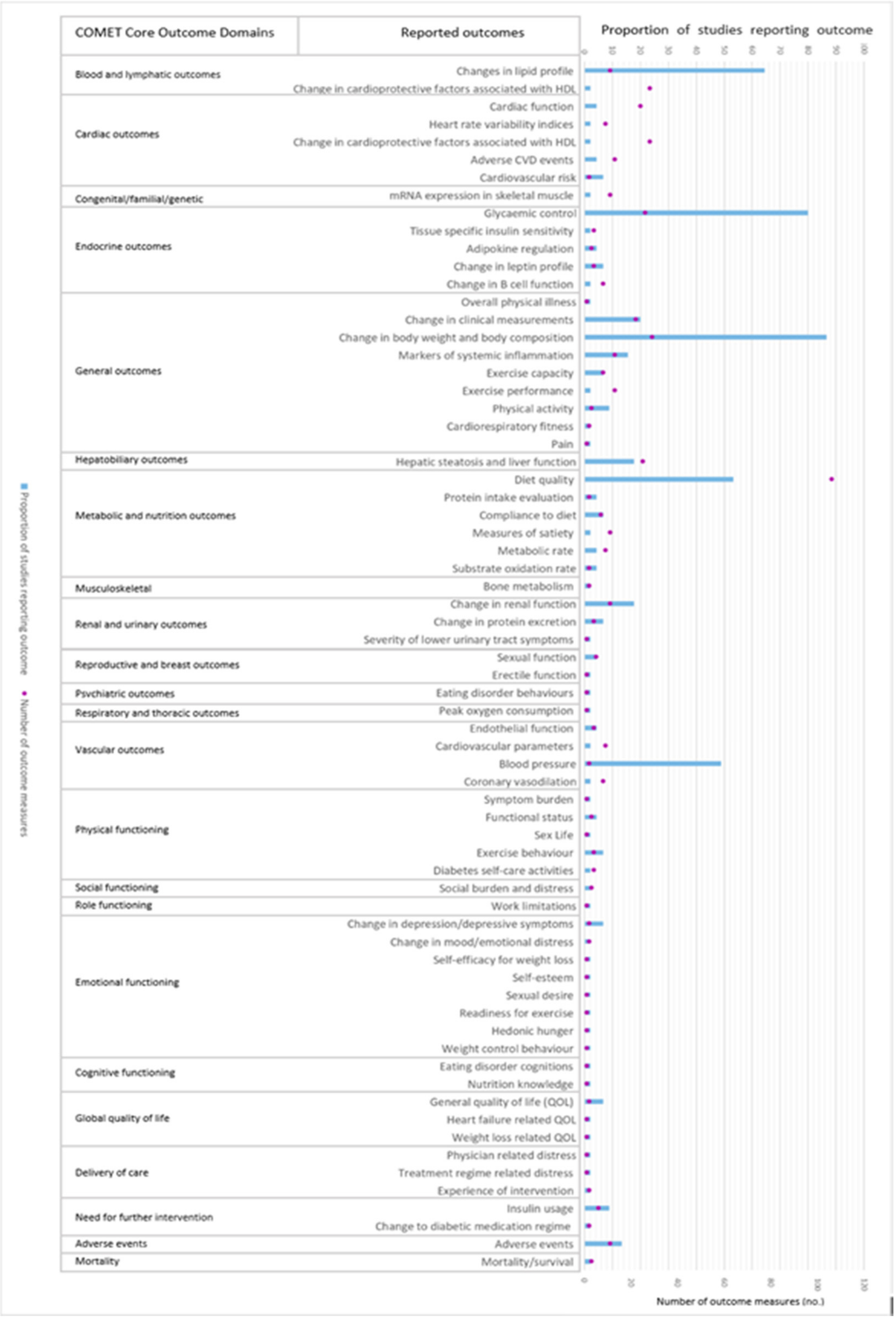

Supplementary Materials Figure S2: Outcome measures for change in body composition reported by RCTs

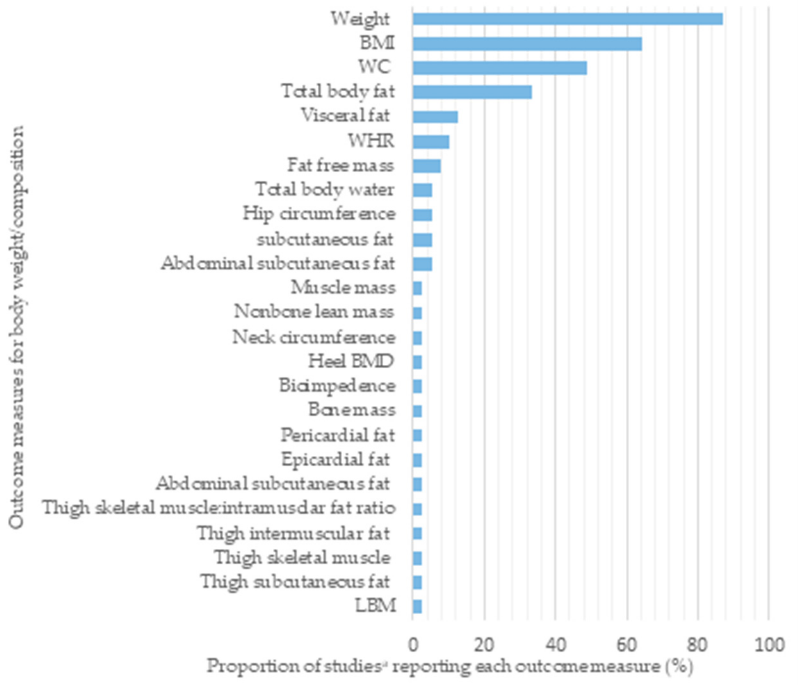

BMI: Body mass index, WC: Waist circumference, WHR: Waist to hip ratio, heel BMD: heel bone mineral density, [a]Only inclusive of studies reporting body weight/composition as an outcome (n=39)

Supplementary Material Figure S3: Outcome measures for glycaemic control reported by RCTs

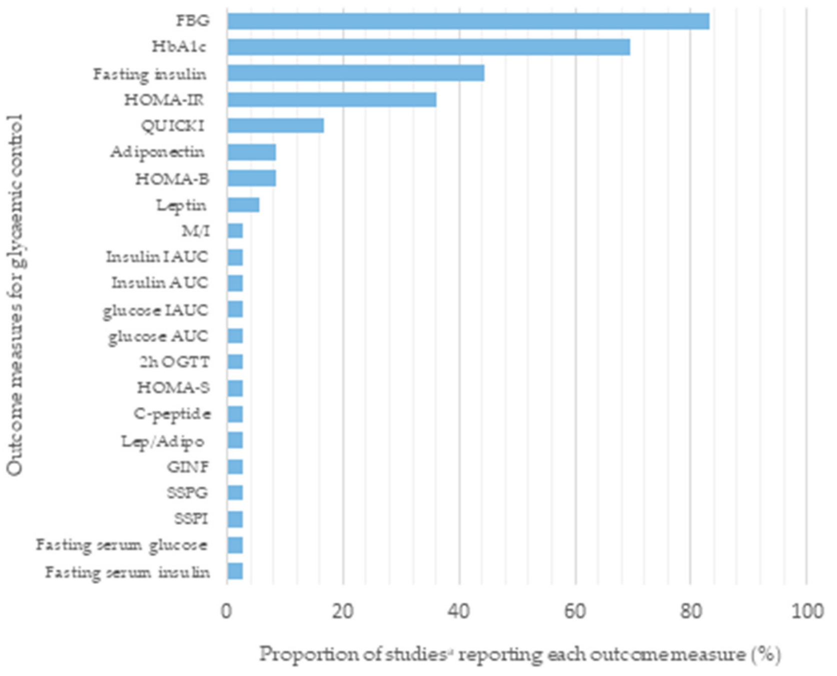

FBG: fasting blood glucose, HbA1c: glycosylated haemoglobin, HOMA-IR: Homeostatic Model Assessment of Insulin Resistance, QUICKI: quantitative insulin-sensitivity check index, HOMA-B: Homeostatic model assessment of β-cell function, M/I: ratio of glucose infusion rate (GINF) to mean steady state insulin, 2h OGTT: two hour oral glucose tolerance test, HOMA-S: insulin sensitivity, lep/adipo: leptin to adiponection ratio, GINF: glucose infusion rate, SSPG: steady-state plasma glucose level, SSPI: steady-state plasma insulin level, [a] Only inclusive of studies reporting 'glycaemic control' as an outcome (n=36)

Supplementary Material Figure S4: Outcome measures for change in lipid profile reported by RCTs

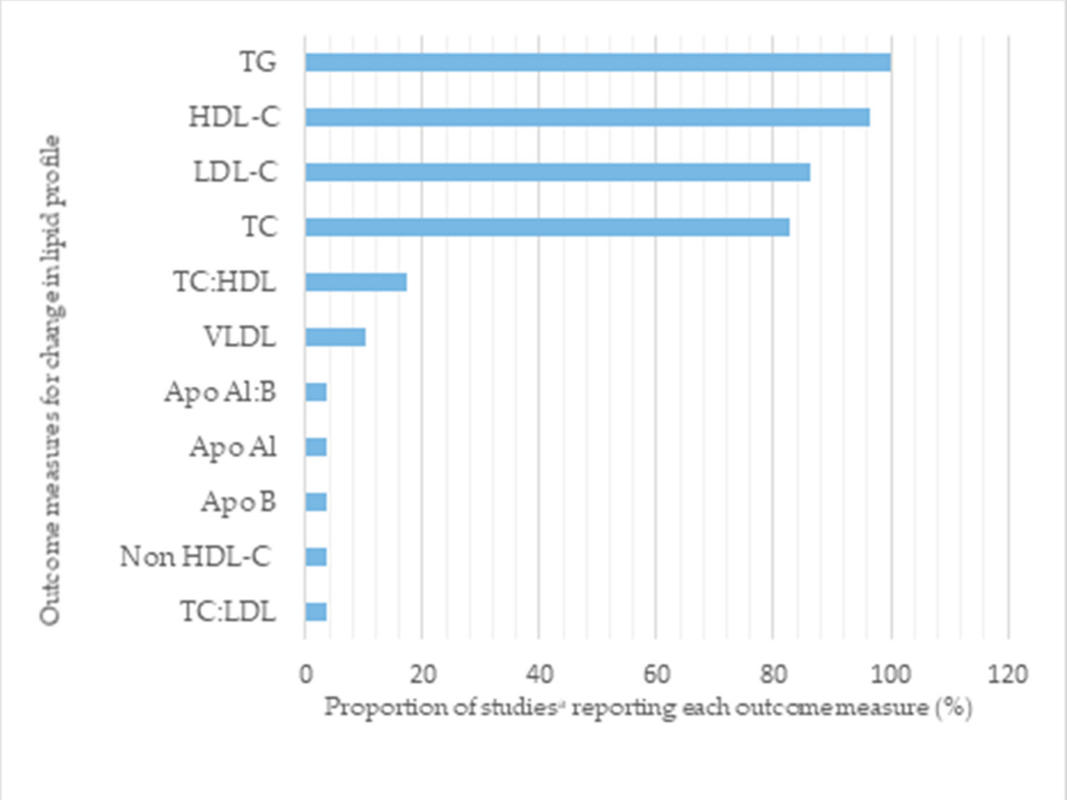

Supplementary Material Figure S5: Outcome measures for change in dietary intake reported by RCTs

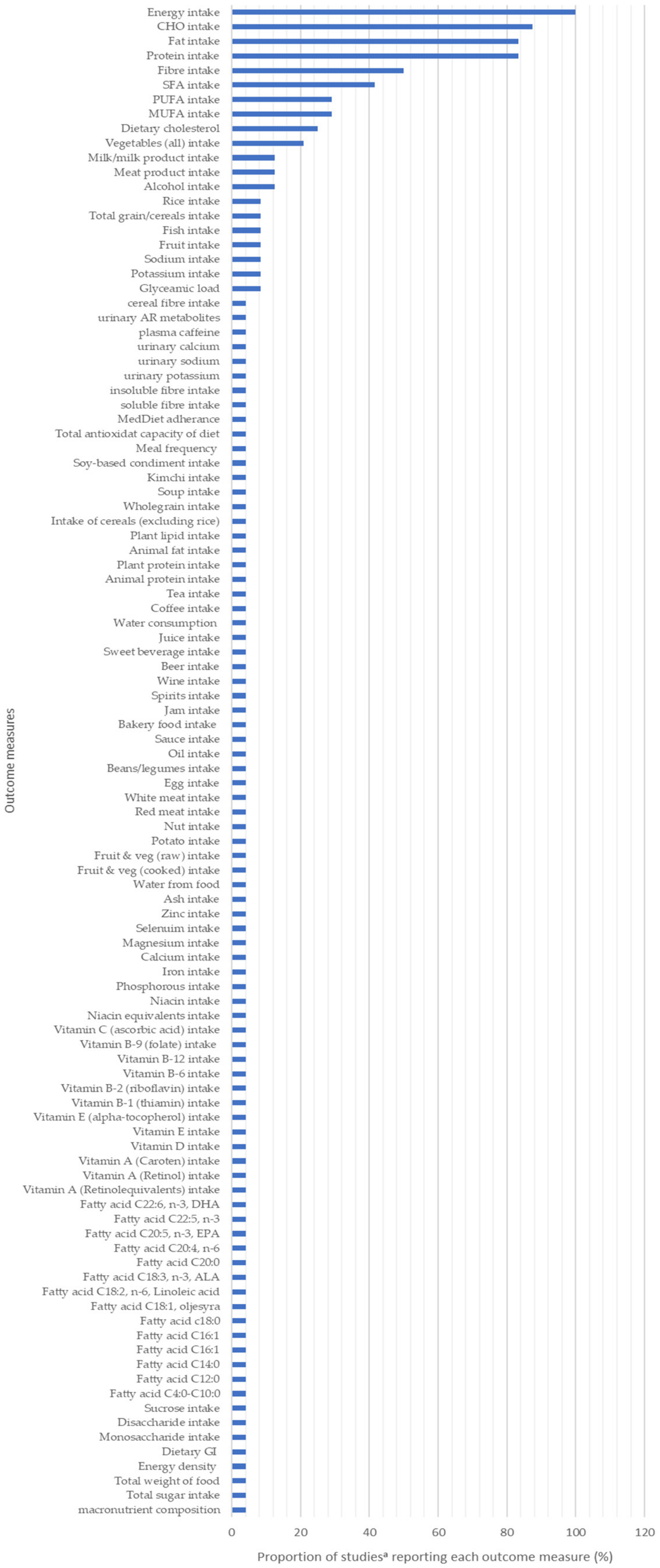

CHO: carbohydrate, SFA: saturated fatty acids, MUFA: monounsaturated fatty acids, PUFA: polyunsaturated fatty acids, [a]Only includes those studies reporting dietary intake as an outcome (n=26)

Supplementary Material Figure S6: Quality assessment of RCTs using Cochrane Risk of Bias 2.0 Tool

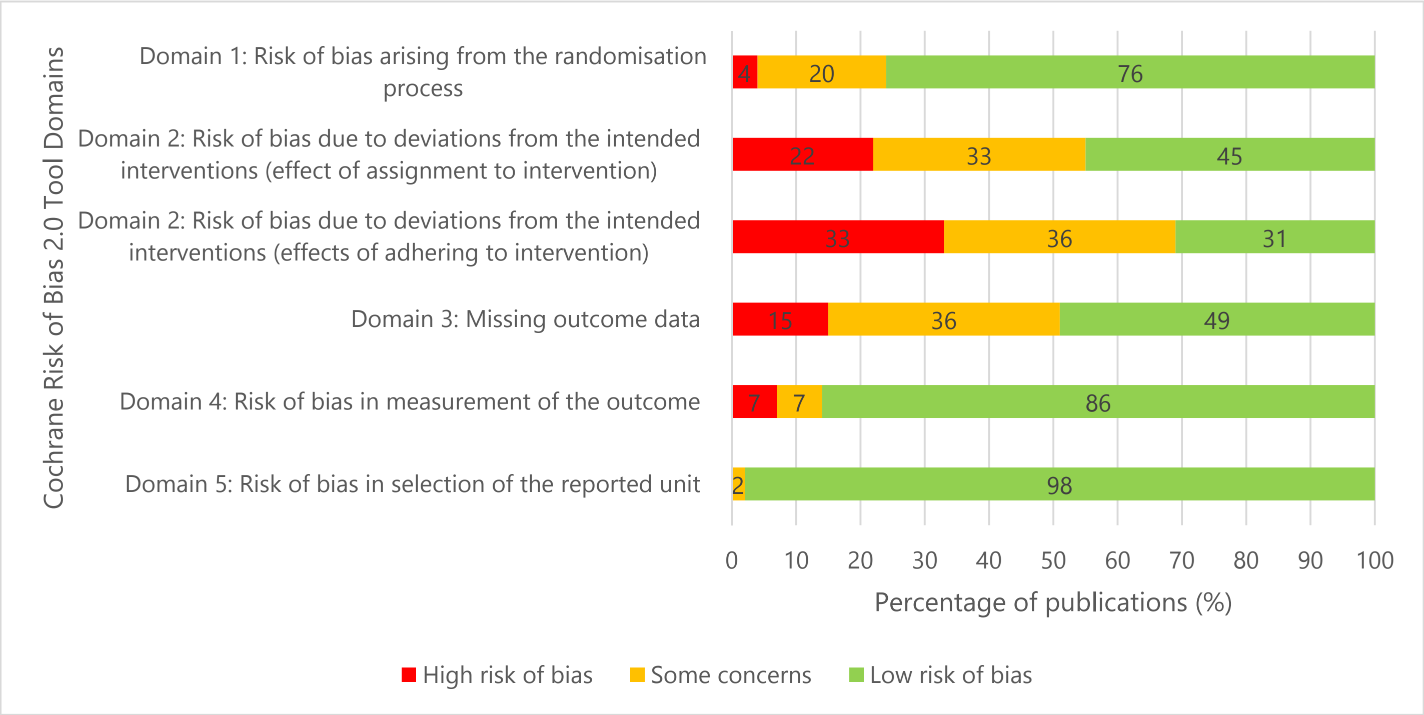

Supplement: Supplementary file 1 [file nutrients-13-03388-s001.zip › nutrients-1380292-supplementary.pdf]
